# Gut Microbiota Signature Among Asian Post-gestational Diabetes Women Linked to Macronutrient Intakes and Metabolic Phenotypes

**DOI:** 10.3389/fmicb.2021.680622

**Published:** 2021-06-25

**Authors:** Zubaidah Hasain, Raja Affendi Raja Ali, Shairah Abdul Razak, Kamalrul Azlan Azizan, Emad El-Omar, Nurul Huda Razalli, Norfilza Mohd Mokhtar

**Affiliations:** ^1^Department of Physiology, Faculty of Medicine, Universiti Kebangsaan Malaysia, Kuala Lumpur, Malaysia; ^2^Gastroenterology Unit, Department of Medicine, Faculty of Medicine, Universiti Kebangsaan Malaysia, Kuala Lumpur, Malaysia; ^3^GUT Research Group, Faculty of Medicine, Universiti Kebangsaan Malaysia, Kuala Lumpur, Malaysia; ^4^Department of Applied Physics, Faculty of Science and Technology, Universiti Kebangsaan Malaysia, Bangi, Malaysia; ^5^Metabolomics Research Laboratory, Institute of Systems Biology, Universiti Kebangsaan Malaysia, Bangi, Malaysia; ^6^Microbiome Research Centre, St George and Sutherland Clinical School, University of New South Wales, Sydney, NSW, Australia; ^7^Dietetic Program, Faculty of Health Sciences, Universiti Kebangsaan Malaysia, Kuala Lumpur, Malaysia

**Keywords:** gut microbiota, diet, obesity, insulin resistance, gestational diabetes

## Abstract

Aberrant gut microbiota dysbiosis in women with a previous history of gestational diabetes mellitus (post-GDM) was comparable to that in adults with type 2 diabetes mellitus (T2DM). Nonetheless, potential relationships between diet, gut microbiota, and metabolic phenotypes in post-GDM women after delivery are yet to be discovered. In this research, we assessed the relationship of the macronutrient intakes, gut microbiota composition, and metabolic phenotypes (i.e., anthropometrics and glycemic control) in post-GDM women with and without postpartum glucose intolerance (GI). About 24 post-GDM women were included in this study, 14 women were grouped in the GI group and 10 women were grouped in the normal glucose tolerance (NGT) group according to oral glucose tolerance test. Macronutrient intake assessment using a 3-day dietary record, anthropometric measurements, biochemical analyses, and fecal sampling were done during 3–6 months postpartum. Gut microbiota profiling was determined using 16S rRNA genes sequencing targeting the V3–V4 regions. The relationships between macronutrient intakes, gut microbiota composition, and metabolic phenotypes were evaluated using Pearson’s correlation coefficient and stepwise regression analyses. In this study, most post-GDM women had significantly poor dietary fiber adherence than the nutritional recommendations. Women from the GI group have significantly higher fasting blood glucose (FBG), HbA1c, and homeostasis model assessment-estimated insulin resistance (HOMA-IR) levels compared to the NGT group. The group also showed significant elevation of high-sensitivity C-reactive protein (hs-CRP) level when compared to the normal value. Specific gut microbial taxa derived from Proteobacteria and Bacteroidetes such as *Parasutterella, Aquicella, Haliscomenobacter*, and *Prevotellaceae*_NK3B31_group were significantly abundant in the GI group compared to the NGT group. *Prevotellaceae_*NK3B31*_*group was significantly associated with high FBG, HOMA-IR, and HbA1c levels. Low fiber and monounsaturated fatty acids intakes were associated with *Lactobacillus*. Meanwhile, *Lactobacillus* was associated with high body mass index, waist circumference, 2-h postprandial blood glucose, and hs-CRP levels. Our study suggested that macronutrient intake is an important predictor of gut microbiota dysbiosis and is associated with obesity, low-grade inflammation, and poor glycemic control in post-GDM women. Hence, dietary intake modification to remodel gut microbiota composition is a promising T2DM preventive strategy in post-GDM women.

## Introduction

Gestational diabetes mellitus (GDM) is a common pregnancy complication and associated with 7-fold higher risk of developing type 2 diabetes mellitus (T2DM) later in life compared to healthy women (Bellamy et al., 2009). The relative risk of T2DM in women with a previous history of GDM varies across countries, ranging from 2.1 in Germany to 47.3 in the United States (Zhu and Zhang, 2016). Biomarkers are important to predict the risk of T2DM in post-GDM women at an early stage because the progression of GDM to T2DM is an emerging public health concern. Interestingly, current ongoing studies are revealing possible metabolic cross-talk between gut microbiota and GDM. Perturbation of normal gut microbiota composition or gut microbiota dysbiosis, may cause host metabolism dysregulation and is responsible for numerous diseases, including GDM (Hasain et al., 2020). Mokkala et al. (2017) reported that the composition of Ruminococcaceae in the first trimester was higher and preceded the diagnosis of GDM compared to healthy pregnant women (Mokkala et al., 2017). Meanwhile, Kuang et al. (2017) found several gut microbiotas, including *Parabacteroides distasonis*, *Klebsiella variicola*, and *Eubacterium rectale*, were associated with maternal glucose levels in mothers with GDM (Kuang et al., 2017). On the other hand, the amount of beneficial butyrate-producing bacteria, such as *Bifidobacterium* and *Faecalibacterium*, were found to be lower in women with GDM than in normoglycemic pregnant women (Kuang et al., 2017; Wang et al., 2018). These findings suggest that gut microbiota could serve as a potential predictive biomarker of glucose intolerance (GI).

The study of gut microbiota in post-GDM women is less explored. The results reported in the limited number of studies were contradictory (Fugmann et al., 2015; Crusell et al., 2018; Hasan et al., 2018; Hasain et al., 2019). Crusell et al. (2018) found the gut microbiota composition in women with GDM during the third trimester of pregnancy and 8 months postpartum was consistent. The gut microbiota composition was dominated by Firmicutes and Actinobacteria (Crusell et al., 2018). However, other studies have reported the relative abundance of Firmicutes in post-GDM women was either reduced (Fugmann et al., 2015; Hasain et al., 2019) or no different (Hasan et al., 2018) compared to the normoglycemic postpartum women. The available gut microbiota studies involving women with GDM have mostly been from Finland, China, and Germany (Hasain et al., 2020). These findings generally represent an urbanized population and may not resemble the universal gut microbiota profile of women with GDM (Gupta et al., 2017). It is necessary to study gut microbiota composition in different geographical locations and populations, as the profile of the dominant gut microbiota such as Bacteroidetes, Firmicutes, Actinobacteria, and Proteobacteria was influenced by cultural and behavioral features (Gupta et al., 2017; Senghor et al., 2018).

Among the environmental factors, a person’s dietary pattern is the most important factor that ensures a healthy gut (Lazar et al., 2019). During pregnancy, a low-fiber diet was reported to be associated with a higher abundance of *Sutterella* and *Collinsella* (Ponzo et al., 2019). *Sutterella* is a genus that was associated with pro-inflammatory properties while *Collinsella* is a genus that was associated with T2DM (Ponzo et al., 2019). Moreover, total fat intake was associated with a greater abundance of *Alistipes* while protein intake was associated with a greater abundance of *Faecalibacterium* among women with GDM during pregnancy (Ferrocino et al., 2018). Following delivery, changing postpartum dietary habits is common, and the intakes could be influenced by over-cautious, special, and traditional dietary practices, particularly among Asian women (Mohd Yusoff et al., 2018). While diet is well known to be linked to gut microbiota composition during pregnancy, more studies are warranted to better understand the dynamics of gut microbiota composition following changes in postpartum dietary intakes among women with histories of GDM.

The aim of this study was to assess the macronutrient intakes, anthropometrics, and glycemic control, as well as evaluate their associations with gut microbiota composition in post-GDM women with and without postpartum GI. We hypothesized that the imbalance in macronutrient intakes during the postpartum period was associated with gut microbiota dysbiosis in post-GDM women. Furthermore, this phenomenon may be an important contributor to postpartum GI, and predispose women with GDM to develop T2DM in the future.

## Materials and Methods

### Study Design and Population

This cross-sectional study was designed to determine the macronutrient intakes, anthropometrics, glycemic control, and their associations with gut microbiota composition of postpartum gestational diabetes women with and without GI. The human ethics approval clearance was approved by the Universiti Kebangsaan’s Malaysia Human Research Ethics Committee (UKM PPI/111/8/JEP-2018-022) for a 2-year period, from March 2018 to February 2020. Approximately 35 postpartum women with a recent history of GDM were recruited at 6–8 weeks postpartum using a systematic random sampling method from the Universiti Kebangsaan’s Malaysia Medical Centre (UKMMC) delivery records. After eligibility screening, this study included only 24 of 35 post-GDM women without pre-pregnancy diabetes, autoimmune diseases, and chronic diseases. The inclusion criteria were strict because this study focusing on postpartum GI following a recent episode of GDM during pregnancy. Notably, possible modulators of the gut microbiota composition such as post-GDM women on regular treatment, including diabetic therapy, anti-inflammatory drugs, laxatives, traditional medicine, or antibiotics/probiotics within 3 months prior to the present study were excluded. Eleven post-GDM women were excluded as they were not willing to participate and did not meet the inclusion criteria. A detailed explanation of the study protocol was provided to participants, and written informed consent was obtained prior to their enrollment. The first examination was performed during 6–8 weeks postpartum to evaluate the participant’s glucose tolerance status. Following childbirth, Malaysian women usually adhere to strict dietary restrictions during the confinement period, especially during the initial 30–60 days after delivery to maintain well-being, and they gradually transition back to their normal diet (Mohd Yusoff et al., 2018). Thus, macronutrient intakes evaluation, anthropometric measurements, biochemical analyses, and fecal sampling for 16S rRNA sequencing were obtained during the second examination (between 3 and 6 months postpartum). Moreover, lifestyle recommendations were not given to eliminate the possible confounding factor. A detailed flowchart of the study is summarized in the supplementary material (Supplementary Figure 1).

### Postpartum Glucose Tolerance Assessment

All participants who met the inclusion criteria provided their socio-demographic information and data related to obstetric history and were then screened for glucose tolerance using a 2-h 75 g oral glucose tolerance test (OGTT). This assessment was done during 6–8 weeks postpartum and considered as the first examination. Fasting blood glucose (FBG) samples were taken after a 10-h overnight fast while 2-h postprandial blood glucose (2HPP) samples were taken after 2-h of consumption of 75 g glucose solution. Plasma glucose levels were determined with hexokinase and glucose-6-phosphate dehydrogenase enzymes using the Slein method on a Siemens ADVIA 2400 (Siemens Healthcare Diagnostics, NY, United States). Characterization of postpartum glucose tolerance was based on the Malaysian Clinical Practice Guidelines (CPG) on Management of T2DM, 5th edition, 2015 (Ministry of Health Malaysia, 2015). Participants exhibiting an FBG level ≥6.1 mmol/L and/or a 2HPP level ≥7.8 mmol/L were designated as the GI group, while participants with an FBG level less than 6.1 mmol/L and a 2HPP level less than 7.8 mmol/L were designated as the normal glucose tolerance (NGT) group (Chew et al., 2012).

### Macronutrient Intake Evaluation

Participants’ macronutrient intakes were assessed once during the second examination (between 3 and 6 months postpartum) using a 3-day dietary record (two weekdays and one weekend). Dietary instructions were explained to the participants using the food photograph and household measures to aid the participants to record their dietary intake accurately. Detail of the macronutrient intakes was calculated using Nutritionist Pro^TM^ Diet Analysis 4.0 (Axxya Systems, Woodinville, WA, United States) software. The proportion of macronutrients was compared with Malaysian dietary guidelines (Ministry of Health Malaysia, 2015; National Coordinating Committee on Food and Nutrition, 2017).

### Anthropometric Measurements

Pre-pregnancy body weight was self-reported, and the height detail was obtained from the antenatal record book. The postpartum anthropometric measurements were evaluated once during the second examination as stated above. Postpartum body weight was measured to the nearest 0.1 kg using a digital scale (SECA, Hamburg, Germany). Body mass index (BMI) was calculated by dividing participants’ body weight (kg) by the square of their height (m^2^). Waist and hip circumferences were measured at the umbilicus and trochanter level, respectively, to the nearest 0.1 cm using a flexible measuring tape. Measurements were repeated twice and the mean of these measurements was calculated to increase the accuracy (Chew et al., 2012). Participants’ waist-to-hip ratios (WHRs) were obtained as follows: waist divided by hip measurements. Classification of BMI, cut-off points for waist circumference, and WHR was according to the Malaysian CPG on the Management of Obesity, 2004 (Ministry of Health Malaysia, 2004). Postpartum weight retention was calculated by measuring the difference between participants’ weights taken at the second examination and their pre-pregnancy weights.

### Glycemic Control Analyses

Glycemic control-related markers, including FBG, glycosylated hemoglobin (HbA1c), insulin, total cholesterol, triglycerides, and high-sensitivity C-reactive proteins (hs-CRP), were assessed once during the second examination as stated above. FBG was measured using the hexokinase method with an intra-assay of 1.2–1.8%, while HbA1c was measured using a turbidimetric inhibition immunoassay (TINIA) method on a Roche Cobas 513 (Roche Diagnostics GmbH, Mannheim, Germany) with an intra-assay of 1.0–1.6%. Insulin was analyzed with a Human Metabolic Hormone Magnetic Bead Panel-Metabolism Multiplex Assay using a MILLIPLEX^®^ MAP kit according to the manufacturer’s guidelines (Merck KGaA, Darmstadt, Germany). Insulin resistance was calculated using the homeostasis model assessment-estimated insulin resistance (HOMA-IR) index as follows: HOMA-IR = fasting insulin (μlU/ml) × FBG (mmol/L)/22.5 (Lee et al., 2016). Total cholesterol levels were determined with an enzymatic assay using cholesterol esterase and cholesterol oxidase conversion, followed by Trinder endpoint with an intra-assay of 0.8–1.1%. Triglyceride levels were analyzed using fosatti three-step enzymatic with Trinder endpoint with an intra-assay of 0.8–1.5%, while hs-CRP values were determined by latex-enhanced turbidimetric/immune-turbidimetric assay with an intra-assay of 1.0–8.2%. FBG, total cholesterol, triglycerides, and hs-CRP were evaluated on a Siemens ADVIA 2400 (Siemens Healthcare Diagnostics, NY, United States).

### Gut Microbiota Evaluation Using 16S rRNA Sequencing Approach

#### Fecal Sample Collection and DNA Extraction

The fecal sample was self-collected at home during the second examination as stated above using a sterilized fecal collection kit and stored at 4°C. A single stool sample was collected per participant. The sample was transported to the laboratory in an icebox within 2-h of collection and immediately stored at −80°C until DNA extraction. Total genome DNA was extracted from 500 mg of the fecal sample using a Fast DNA^TM^ SPIN Kit for soil (MP Biomedical, United States) in accordance with the manufacturer’s guidelines and kept frozen at −20°C until used. A NanoDrop spectrophotometer (Thermo Fisher Scientific, Waltham, MA, United States) and electrophoresis were used to assess the quantity and quality of the total DNA.

#### 16S rRNA Amplicons Generation, Library Preparation, and Sequencing

The 16S rRNA genes targeting the V3–V4 regions were amplified using the barcoded 341F/806R primer pair (341F: 5′-CCT AYG GGR BGC ASC AG-3′; 806R: 5′-GGA CTA CNN GGG TAT CTA AT-3′) (Caporaso et al., 2011). PCR reactions were performed using Phusion^®^ High-Fidelity PCR Master Mix (New England Bio Labs). PCR amplicons bands between 400 and 450 bp were validated as containing qualitatively sufficient bacterial DNA. These PCR amplicons were then mixed in equidensity ratios and purified with a Qiagen gel extraction kit (Qiagen, Germany). Library preparation was performed using a NEBNext^®^ Ultra^TM^ DNA Library Prep Kit (New England Bio Labs, United States). The library quality was checked using a Qubit 2.0 fluorometer (Life Technologies, Carlsbad, CA, United States) and Q-PCR. Finally, the library sequencing was conducted with the Illumina HiSeq2500 platform to generate 250 bp paired-end raw reads. All procedures were performed by Novogene Co., Ltd., located in Beijing, China^1^.

#### Sequence Processing and Bioinformatics Analysis

Sequence data were processed using the default pipeline and computed workflow. First, paired-end sequence merging was performed using FLASH (Magoè and Salzberg, 2011), followed by quality filtering according to QIIME to obtain high-quality clean tags (Caporaso et al., 2010; Bokulich et al., 2013). The UCHIME algorithm was used for chimera assessment and removal (Edgar et al., 2011; Haas et al., 2011). Further sequence analyses were conducted using UPARSE for taxonomic assignments of operational taxonomic unit (OTU) selection based on the 97% sequence’s similarity (Edgar, 2013). Alignments were performed using SILVA-based bacterial reference database (Silva release 128) for each OTU representative sequence using a protocol within an open-source workflow implemented by *mothur* (Quast et al., 2013). OTUs abundance data was later normalized using a standard of sequence number corresponding to the sample with the lowest number of sequences per sample (i.e., 60,960 reads/sample). Rarefaction analyses were performed to evaluate the sampling coverage for each sample based on the selected sequence depth.

#### Alpha Diversity and Beta Diversity

Subsequent analysis of alpha (α) and beta (β) diversity were all performed based on the rarified data. All measures of community diversity and similarity, including Shannon, Chao 1, Phylogenetic Diversity (PD_whole_tree), and Simpson’s diversity indices were calculated from the sequence data within QIIME to quantify α diversity. Comparative community compositional analyses of β diversity were performed to evaluate differences of samples in their species complexity. The estimates of weighted and unweighted UniFrac distances among samples were first calculated by QIIME software (Version 1.7.0). These estimates were later subjected to downstream analyses using several packages implemented in R software (version 2.15.3). The FactoMineR and ggplot2 packages (version 2.15.3) were used for clustering analysis *via* the hierarchical clustering method. Multidimensional reduction and principal coordinate analysis (PCoA) ordination plots were generated to visually compare gut microbiota composition between samples from different participants and their groupings based on the two largest eigenvalues. PCoA analysis was displayed by WGCNA package, stat packages, and ggplot2 package in R software (version 2.15.3).

### Statistical Analysis of Participant’s Characteristics and Gut Microbiota Profile

Comparison of participant characteristics (socio-demographic data, obstetrics data, macronutrient intakes, anthropometric measurements, and glycemic control parameters) between GI and NGT groups were analyzed using SPSS software, version 23 (SPSS, Chicago, IL, United States). The distribution of the data was tested based on skewness and kurtosis. *t*-tests were used to determine the difference of normally distributed continuous data while Mann–Whitney tests were used to investigate the difference of not normally distributed continuous data between the two groups. The Pearson’s Chi-square test was used to test the difference of categorical data between the two groups. Comparison of macronutrient intakes and glycemic control parameters with the recommended value was performed using a one-sample *t*-test for normally distributed data and sign test for not normally distributed data. *p*-value < 0.05, *p*-value < 0.01, and *p*-value < 0.001 were considered to be statistically significant and denoted as ^∗^, ^∗∗^, and ^∗∗∗^, respectively.

Comparison of the top 10 relative abundance of the gut microbiota at the phylum and genus level between both groups was visualized as stacked bars of each participant using GraphPad Prism software, version 8.0.2. To test for significant differences in α indices of gut microbiota between different glucose tolerance groups (GI vs NGT), samples were analyzed using a non-parametric Wilcoxon test. A *p*-value below 0.05 indicated significant differences in pairwise mean comparisons. The β diversity measurement by multivariate hypothesis testing was conducted using Adonis functions in the vegan package. OTUs with the highest correlations with the PCoA x and y component axes were identified based on Pearson’s correlation coefficients using the *corr* function.

Further statistical analysis to distinguish gut microbiota communities between GI and NGT groups was performed by linear discriminant analysis (LDA) combined with effect size measurements (LEfSe). Using LEfSe software, the LDA scores’ histogram presents species (from the phylum down to the genus level) whose abundance shows significant differences between the two groups (Segata et al., 2011). The α-value for the pairwise Wilcoxon test was set at 0.05, while the threshold on the logarithmic LDA score for discriminative features was set at 2.0. Besides, *metastats* were used to determine the gut microbial taxa that statistically different between the two groups based on their abundance. *Metastats* analysis is a strict statistical method where the *p*-value was calculated using the permutation test method while the *q*-value was calculated by the method of Benjamini and Hochberg false discovery rate (FDR) (White et al., 2009). *Metastats* results with a *q*-value less than 0.5 were considered to be statistically significant. All analyses were performed in the programming and statistical software R (version 2.15.3).

The strength of relationships between gut microbiota composition and macronutrient intakes, anthropometrics, and glycemic control were evaluated using Pearson’s correlation coefficient analysis and visualized using a heat map. All relevant parameters were logged transformed, and Pareto scaled k-nearest neighbor (KNN) algorithm was used to estimate missing values. Macronutrient intake, anthropometric, and glycemic control parameters correlated with the relative abundance of the 60 gut microbial taxa. Only 60 gut microbial taxa at the genus level were selected as the rest of the OTU data were excluded from the OTU data analysis, which contained >50% missing values. Further analysis to test the significant influence between these parameters and the gut microbial taxa was assessed using stepwise linear regression analysis. Evaluation of the association between the predictor and dependent variables showed better predictions when the combinations of predictors were chosen through stepwise methods. Association of macronutrient intakes and gut microbial taxa was performed by selecting macronutrient intake parameters as the predictor and gut microbial taxa as the dependent variable. By contrast, the association of gut microbial taxa with anthropometric and glycemic control parameters was performed by selecting gut microbial taxa as the predictor while the anthropometric and glycemic control parameters were selected as dependent variables. Both Pearson’s correlation coefficient and stepwise linear regression analyses were performed using SPSS version 23. *p*-value < 0.05, *p*-value < 0.01, and *p*-value < 0.001 were considered to be statistically significant and denoted as ^∗^, ^∗∗^, and ^∗∗∗^, respectively.

## Results

### Characteristics of the Participants

The characteristics of the post-GDM women are summarized in Table 1 and Supplementary Table 1. Twenty-four post-GDM women participated in this study and were grouped based on the postpartum glucose tolerance test; 14 women were grouped in the GI group, and ten were grouped in the NGT group. Among 14 post-GDM women from the GI group, eight (57.1%) women have impaired glucose tolerance (IGT), one (7.1%) woman has combined impaired fasting glucose (IFG) and IGT, and five (35.7%) women have diabetes (Table 1). Majority of the participants were above 30 years old, obese, and of Malay ethnicity. The NGT group had a significantly higher education level than the GI group (*p* < 0.05). Pharmacological requirements during pregnancy (i.e., insulin and metformin) were significantly higher in the GI group. Women from the GI group have a lower percentage of exclusive breastfeeding than the NGT group (28.6%). Most of the macronutrient intakes of these women showed no significant difference between the two groups except for protein intakes, which was significantly lower in the GI group than in the NGT group (Table 1). Total cholesterol intakes were higher in the GI group than the NGT group, though it was not statistically significant (Table 1). Based on the anthropometric assessments, the GI group showed greater elevation of postpartum weight gain by 4.95 kg and had higher BMI values, waist circumference, and obesity percentage compared to the NGT group. The FBG and 2HPP glucose levels during OGTT at the first visit assessment, as well as FBG, HbAIc, HOMA-IR, and triglycerides levels during the second visit assessment were significantly higher in the GI group compared to the NGT group (*p* < 0.05) (Table 1). The comparisons of the macronutrient intake and glycemic control parameters of both groups with the recommended value are shown in Supplementary Table 2. Both groups consumed very low total dietary fiber intakes (*p* < 0.001) and higher total cholesterol intakes compared to the Malaysian dietary guidelines (Ministry of Health Malaysia, 2015; National Coordinating Committee on Food and Nutrition, 2017). The rest of the macronutrients were within the recommended range. Meanwhile, the GI group showed a significant elevation of hs-CRP levels than the recommended value. Besides, both groups showed significantly high HOMA-IR compared to the recommended value by the Malaysian CPG on Management of Type 2 Diabetes Mellitus, 5th edition, 2015 (Supplementary Table 2; Ministry of Health Malaysia, 2015).

**TABLE 1:** Characteristics of post-gestational diabetes mellitus women categorized by postpartum glucose tolerance.

Characteristics	Glucose intolerance (*N* = 14)	Normal glucose tolerance (*N* = 10)	*p*-Value
Age (years)	33.71 ± 3.83	35.90 ± 3.54	0.169
Pre-pregnancy BMI (kg/m^2^)	29.18 ± 5.65	29.49 ± 5.20	0.895
Ethnicity [*n* (%)]			
Malay	12 (85.7)	10 (100)	0.212^##^
Others	2 (14.3)	0 (0)	
Education level [*n* (%)]			
Secondary	7 (50.0)	1 (10.0)	0.040^##^*
Tertiary	7 (50.0)	9 (90.0)	
Number of children	3 ± 1	3 ± 1	0.212
Family history of T2DM [*n* (%)]	12 (85.7)	10 (100)	0.170^##^
Pharmacological requirement during pregnancy [*n* (%)]	10 (71.4)	3 (30.0)	0.045^##^*
Exclusive breast feeding [*n* (%)]	4 (28.6)	5 (50.0)	0.285^##^
**OGTT assessment (6–8 weeks postpartum)**			
FBG (mmol/L)	5.8 ± 0.94	4.68 ± 0.40	0.002**
2HPP (mmol/L)	9.00 (7.88–9.00)	5.65 (4.53–6.48)	<0.001^#^***
**Glucose intolerance classification [*n* (%)]**			
IFG	0 (0)		
IGT	8 (57.1)		
IFG + IGT	1 (7.1)		
T2DM	5 (35.7)		
**Second visit assessment (3–6 months postpartum)**			
Months after delivery (months)	3.64 ± 1.64	4.4 ± 1.26	0.245
**Macronutrient intakes**			
Total energy intakes (kcal/day)	1709 ± 360	1694 ± 163	0.905
Carbohydrate (% total kcal)	52.17 ± 5.09	49.89 ± 5.45	0.305
Fat (% total kcal)	32.25 (27.96–5.88)	32.26 (28.97–35.74)	0.953^#^
Proteins (% total kcal)	15.18 (14.51–16.46)	19.00 (16.12–20.06)	0.026^#^*
Cholesterol intakes (mg/day)	286.34 (149.94–335.33)	200.66 (126.49–369.98)	0.770^#^
Saturated fat (g/day)	10.60 ± 4.35	10.12 ± 3.56	0.776
Monounsaturated fat (g/day)	9.55 ± 3.83	11.37 ± 5.25	0.335
Polyunsaturated fat (g/day)	8.62 ± 3.23	9.07 ± 3.74	0.752
Total sugar (g/day)	24.73 (20.93–37.27)	24.09 (15.36–45.29)	0.770^#^
Total dietary fiber (g/day)	4.79 (2.46–6.57)	5.12 (3.66–11.91)	0.364^#^
**Anthropometric measurements**			
Weight gain (kg)	4.65 ± 2.57	0.81 ± 5.80	0.075
BMI (kg/m^2^)	31.02 ± 6.29	29.95 ± 4.41	0.650
Waist circumference (cm)	94.54 ± 11.79	91.95 ± 8.03	0.555
Waist to hip ratio	0.85 ± 0.05	0.84 ± 0.06	0.789
Obesity [*n* (%)]	10 (71.4)	6 (60.0)	0.214^##^
**Glycemic control parameters**			
FBG (mmol/L)	6.35(5.18–7.40)	4.65(4.25–5.05)	0.004^#^**
HbAIc (%)	5.95(5.48–6.78)	5.20(5.00–5.30)	0.009^#^**
Fasting insulin (μlU/ml)	19.62(7.61–47.04)	7.14(0.00–77.33)	0.318^#^
HOMA-IR (mmol/L* μlU/ml)	12.42(10.12–14.47)	9.09(8.31–9.88)	0.001^#^***
Total cholesterol (mmol/L)	4.75(4.45–5.10)	5.05(4.15–6.40)	0.573^#^
Triglycerides (mmol/L)	1.21(0.99–1.61)	0.74(0.64–1.05)	0.021^#^*
hsCRP (mg/L)	6.99 ± 4.75	4.89 ± 5.50	0.329

*Data are presented as median (Q1–Q3), mean ± standard deviation or as *n* (%).*

Significance difference between two groups was calculated with *t*-test except for ^#^Mann–Whitney U test and ^##^Pearson’s Chi-square test.

*p-value < 0.05, **p-value < 0.01, ***p-value < 0.001 were considered as statistically significant.

*BMI, body mass index; T2DM, type 2 diabetes mellitus; OGTT, oral glucose tolerance test; FBG, fasting blood glucose; 2HPP, 2-h postprandial blood glucose; IFG, impaired fasting glucose; IGT, impaired glucose tolerance; HbAIc, glycosylated hemoglobin; HOMA-IR, homeostasis model assessment-estimated insulin resistance; hs-CRP, high-sensitivity C-reactive protein.*

### Sequencing Summary

Rarefaction analyses showed that sequencing efforts were consistent across replicate samples and treatment groups at a depth of 60,960 sequences per sample, as denoted by total percentage coverage higher than 98%. We could sample a large portion of the OTUs and diversity present while still retaining a large number of samples within each group (GI and NGT). After quality filtering, the 16S rRNA amplicon data set produced 2,081,515 high-quality reads. In total, we observed 1593 OTUs defined at 97% sequence identity.

### Gut Microbial Community Composition in the GI and NGT Groups

**FIGURE 1:**
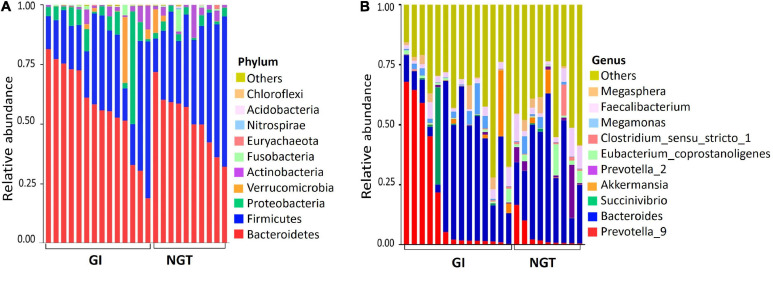
**(A)** Top 10 relative abundance of the gut microbiota at the phylum level between GI and NGT groups. **(B)** Top 10 relative abundance of the gut microbiota at the genus level between GI and NGT groups. Each stacked bar resembles the relative abundance of the gut microbiota of one post-GDM woman. GI, glucose intolerance; NGT, normal glucose tolerance.

The relative abundance of the gut microbiota composition between GI and NGT groups varied at multiple taxonomic levels. General patterns of the top 10 bacterial phylum-level contributions to gut microbiota were shown according to glucose tolerance grouping (Figure 1A). The three most abundant phyla present in both groups were Bacteroidetes, Firmicutes, and Proteobacteria. However, the GI group had a relatively higher abundance of Bacteroidetes and a lower abundance of Firmicutes than in the NGT group. At the genus level, the predominant genera in the GI group included *Prevotella*_9 and *Bacteroides* (Figure 1B). Consistently, further analysis represented by the heat map of the top 56 genera showed the abundance of genera was dominated more by *Prevotella_*9 and *Bacteroides* in the GI group than in the NGT group (Supplementary Figure 2).

### Gut Microbial Alpha Diversity in the GI and NGT Groups

**FIGURE 2:**
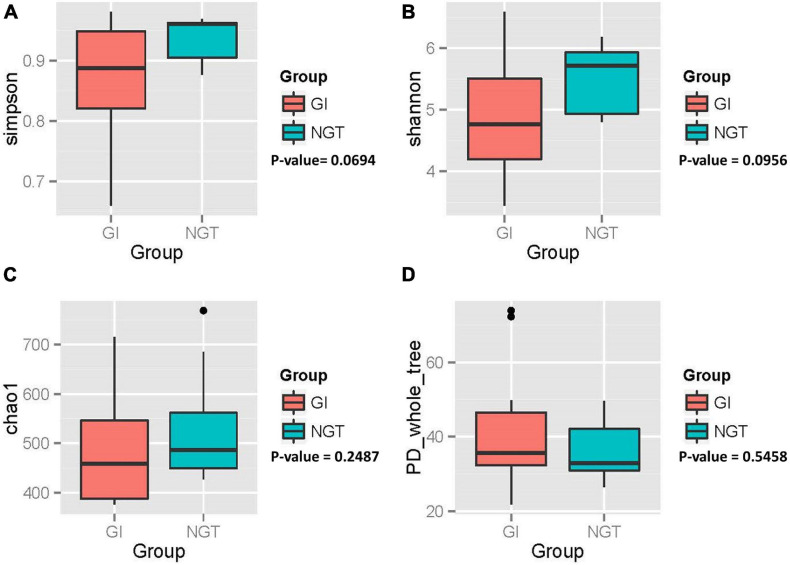
Box plots showing the alpha diversity of the gut microbiota of post-GDM women in the GI and NGT groups. **(A)** Simpson index, **(B)** Shannon index, **(C)** Chao_1 index, and **(D)** PD_whole_tree index. *p*-value obtained from the non-parametric Wilcoxon test. *p*-value < 0.05 was considered as statistically significant. GDM, gestational diabetes mellitus; GI, glucose intolerance; NGT, normal glucose tolerance; PD, phylogenetic diversity.

Variation of the gut microbiota community structure (α diversity) within each woman was evaluated based on species richness (Chao_1 index), evenness (Shannon and Simpson’s index), and phylogenetic diversity (PD_whole_tree index), as shown in Figure 2. Overall, post-GDM women in the GI group exhibited a gut microbial community with lower richness, evenness, and an almost similar phylogenetic diversity compared to the NGT group. However, further analysis using the non-parametric Wilcoxon test showed no significant difference in α diversity between GI and NGT groups (*p* > 0.05).

### Gut Microbial Beta Diversity in the GI and NGT Groups

**FIGURE 3:**
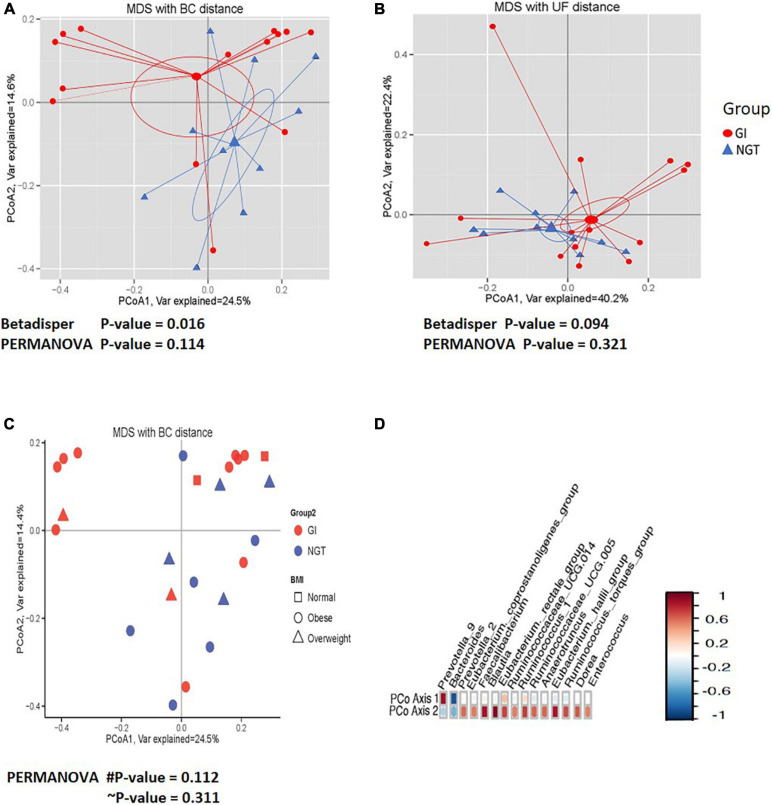
The comparisons of the gut microbiota community (β diversity) between GI (red circle) and NGT (blue) groups. **(A)** Principal coordinate analysis (PCoA) plot based on the OTUs relative abundance using the Bray–Curtis (BC) distance. **(B)** PCoA plot based on the phylogenetic relationship using the UniFrac (UF) distance. The larger shapes are the centroids and linear lines are connected from the samples to the centroid for each group. **(C)** PCoA plot based on the OTUs relative abundance using the BC distance according to the BMI classification [normal (square), overweight (triangle), and obese (circle)] between GI (red circle) and NGT (blue) groups. PCoA1 and PCoA2 account for the horizontal and the vertical variances, respectively. The symbol # indicates *p*-value for comparison between GI and NGT groups. The symbol ∼ indicates *p*-value for comparison between BMI status. *p*-value < 0.05 was considered as statistically significant. **(D)** Correlation plot showing the OTUs with the high correlation with first (PCoA1) and second (PCoA2) axes. The red color indicates a positive correlation and the blue color indicates a negative correlation. GI, glucose intolerance; NGT, normal glucose tolerance; MDS, multidimensional scaling; BMI, body mass index; OTU, operational taxonomic unit.

Next, PCoA analysis based on OTU abundance using Bray–Curtis (BC) distance showed that the ordination pattern of gut microbiota in the participants associated with different glucose tolerance slightly overlapped between the two groups (Figure 3). However, the same ordination analysis on a phylogenetic relationship using the UniFrac (UF) distance indicated that the gut microbiota structure overlapped considerably (Figure 3B). Moreover, hypothesis testing using permutational multivariate analysis of variance (PERMANOVA) for both BC and UF distance did not significantly differ in the centroid of gut microbiota community abundance (β diversity) between GI and NGT groups (Figures 3A,B). Significant dispersion of the gut microbiota across respondents was detected using the betadisper test on the BC distance (*p* < 0.05) from both the NGT and GI groups, though not significant was apparent when tested on the UF distance (Figures 3A,B). We also plotted the ordination of the gut microbiota community based on the BMI status (Figure 3C). However, the gut microbiota community was scattered and deemed comparable between normal, overweight, and obese women (Figure 3C). Interestingly, several numbers of genera, including *Bacteroides*, *Prevotella_9*, *Prevotella_2*, *Faecalibacterium*, *Blautia*, and *(Eubacterium)*_hallii_ groups were found to be highly correlated with the PCoA1 and PCoA2 axes, suggesting their presence might affect the overall gut microbiota composition among the women (Figure 3D).

### Gut Microbiota Signature in the GI Group and NGT Groups

**FIGURE 4:**
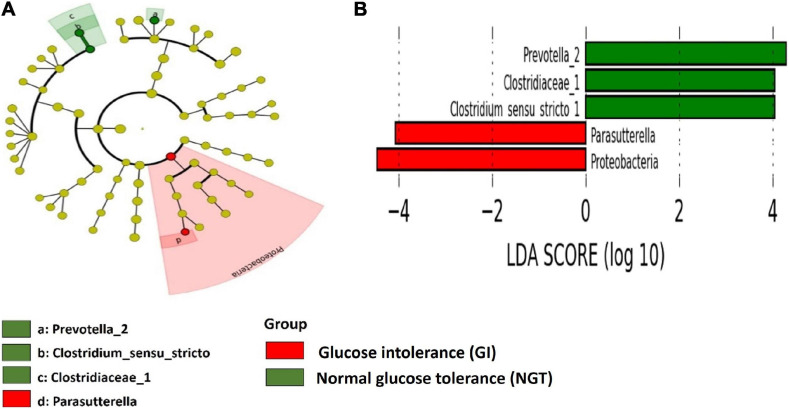
Linear discriminant analysis (LDA) effect size (Lefse) analysis shows differently abundant gut microbial taxa down to genus level between GI and NGT groups. **(A)** Cladogram represents the predominant gut microbial taxa in the GI and the NGT groups. The point in the center of the cladogram reflects the root of the tree (bacteria) while the innermost ring reflects the phylum level. Subsequent outer rings reflect the next taxonomic level (i.e., class, order, family, and genus). **(B)** The histogram represents the LDA score computed for each differently abundant gut microbial taxon between GI and NGT groups. The α-value <0.05 and LDA score ≥2.0 were considered statistically significant.

**FIGURE 5:**
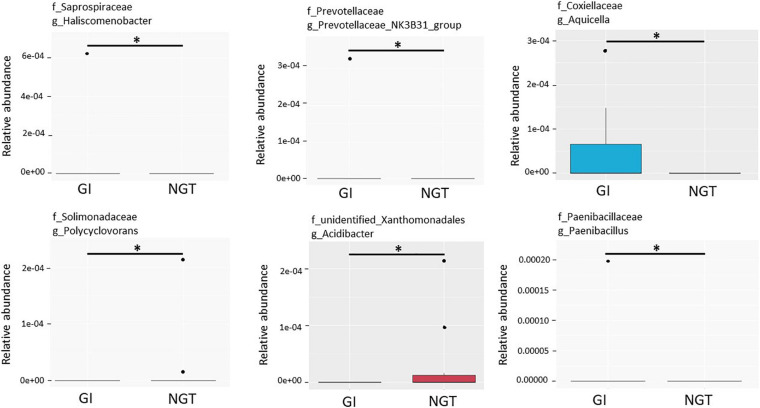
Box plots showing the top six relative abundance of the genera that significantly differed between GI and NGT groups using *metastats* corrected by the Benjamini–Hochberg method. The box represents the interquartile range (IQR), the inside line represents the median, the whisker represents values within 1.5 × IQR of the first and third quartile, and the circle represents the outliers. ^∗^Genera with *q*-value < 0.05 were considered statistically significant. GI, glucose intolerance; NGT, normal glucose tolerance.

LEfSe scores were computed for differentially abundant taxa across both groups (Figure 4). Phylum Proteobacteria and the parent genus *Parasutterella* were identified as the biomarkers for the GDM group, while genus *Prevotella_2*, family Clostridieaceae_1, and genus *Clostridium*_sensu_stricto_1 were the biomarkers for the NGT group (Figure 4). Moreover, several unique gut microbiotas were discovered and differed significantly between GI and NGT groups at multiple levels using *metastats* analysis. At the genus level, *Haliscomenobacter*, *Prevotellaceae_*NK3B31_group, and *Aquicella* were the top genera that were significantly abundant in the women with GI, while *Polycyclovorans* and *Acidibacter* were significantly abundant in the NGT group (Figure 5).

### Correlation of Gut Microbiota Composition With Macronutrient Intakes and Metabolic Phenotypes in Post-GDM Women

**FIGURE 6:**
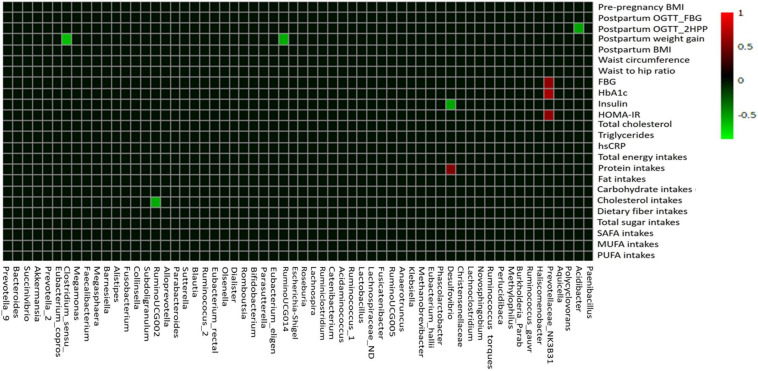
The correlation heat map of the gut microbiota composition with macronutrient intake, anthropometric, and glycemic control variables. Pearson’s correlation coefficient between top 60 gut microbial taxa at the genus level and macronutrient intake, anthropometric, and glycemic control parameters are indicated by colors (red, positive; green, negative). BMI, body mass index; OGTT, oral glucose tolerance test; FBG, fasting blood glucose; 2HPP, 2-h postprandial blood glucose; HbAIc, glycosylated hemoglobin; HOMA-IR, homeostasis model assessment-estimated insulin resistance; hs-CRP, high-sensitivity C-reactive protein; SAFA, saturated fatty acids; PUFA, polyunsaturated fatty acids; MUFA, monounsaturated fatty acids.

Using Pearson’s correlation coefficient analysis, few gut microbial taxa showed moderate correlations (0.5 ≥ *r* ≤ 0.7) and mostly showed weak correlations (0.3 ≥ *r* ≤ 0.49) with the macronutrient intake, anthropometric, and glycemic control parameters (Figure 6 and Supplementary Table 3). Among the macronutrient intakes, protein intakes had moderate positive correlation with relative abundance of *Desulfovibrio* (*r* = 0.542; *p* < 0.01; Figure 6). Comparatively, cholesterol intakes showed moderate negative correlation with relative abundance of *Ruminococcaceae_*UCG002 (*r* = −0.547; *p* < 0.05; Figure 6). Among the gut microbial taxa, we found that *Prevotellaceae*_NK3B31_group had moderate positive correlations with glycemic control parameters, such as HbA1c (*r* = 0.680; *p* < 0.01), FBG (*r* = 0.589; *p* < 0.01), and HOMA-IR levels (*r* = 0.588; *p* < 0.01). On the other hand, *Desulfovibrio* showed negative correlation with fasting insulin levels (*r* = −0.532; *p* < 0.01) while *Acidibacter* negatively correlated with postpartum OGTT_2HPP glucose levels (*r* = −0.530; *p* < 0.01). Moreover, *Clostridum*_sensu_stricto_1 (*r* = −0.612; *p* < 0.01) and *Ruminococcaceae*_UCG014 (*r* = −0.547; *p* < 0.01) showed significant negative correlation with postpartum weight gain (Figure 6 and Supplementary Table 3).

### Association Between Macronutrient Intakes, Gut Microbiota Composition, and Metabolic Phenotypes in Post-GDM Women

**FIGURE 7:**
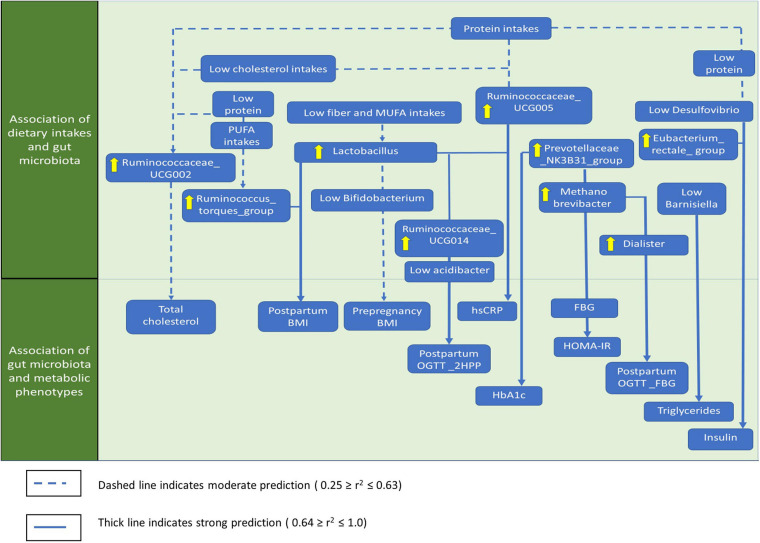
The summary of the genera that significantly associated with macronutrients, anthropometrics, and glycemic control. Only the most significant genera that influence the associations were shown. SAFA, saturated fatty acids; PUFA, polyunsaturated fatty acids; MUFA, monounsaturated fatty acids; BMI, body mass index, WHR, waist to hip ratio; OGTT, oral glucose tolerance test; FBG, fasting blood glucose; 2HPP, 2-h-post prandial blood glucose; HOMA-IR, homeostasis model assessment-estimated insulin resistance; hs-CRP, high-sensitivity C-reactive protein.

The significant associations between macronutrients, gut microbial taxa, anthropometrics, and glycemic control based on stepwise linear regression analysis are summarized in Figure 7 and Supplementary Table 4. Most gut microbiota predictions by macronutrient intakes showed low to moderate significant associations. *Ruminococcaceae_*UCG002 was moderately predicted by protein (linearly) and cholesterol intakes (inversely; *r*^2^ = 0.572, *p* = 0.001). *Phascolarctobacterium* showed lower prediction by fiber (linearly) and total sugar intakes (inversely; *r*^2^ = 0.437, *p* = 0.002). For anthropometrics, postpartum BMI was moderately predicted by *Lactobacillus* and the *Ruminococcus_*torques_ group, though it inversely predicted by *Bifidobacterium*, *Lachnospira*, and *Romboutsia* (*r*^2^ = 0.501, *p* = 0.001). For glycemic control parameters, we identified several strong significant predictions. For instance, a group of gut microbial taxa, including *Prevotellaceae_*NK3B31_group, *Methanobrevibacter*, *Ruminococcaceae_*UCG014 were linearly predicted, while *(Eubacterium)*_hallii__group, *Bacteroides*, and *Acidibacter* were inversely predicted FBG (*r*^2^ = 0.959, *p* = 0.001) and HOMA-IR levels (*r*^2^ = 0.947, *p* = 0.001). Postpartum 2HPP blood glucose was mainly predicted by *Ruminococcaceae*_UCG014 and *Lactobacillus* (*r*^2^ = 0.872, *p* = 0.001). Meanwhile, fasting insulin levels were strongly predicted by the *Eubacterium_*rectale_group and inversely predicted by *Desulfovibrio*, *Faecalibacterium*, and *Catenibacterium* (*r*^2^ = 0.760, *p* = 0.01). Triglycerides levels were inversely predicted by *Barnesiella*, *Alloprevotella*, *Dialister*, and *Acidibacter* but were linearly predicted by *Catenibacterium* (*r*^2^ = 0.750, *p* < 0.001). Postpartum OGTT_FBG was linearly predicted by *Dialister* and *Methanobrevibacter* (*r*^2^ = 0.600, *p* = 0.001). Besides, hs-CRP levels were predicted linearly by *Lactobacillus* and inversely by *Ruminococcaceae_*UCG014 (*r*^2^ = 0.566, *p* = 0.001). *Ruminococcaceae*_UCG002 linearly predicted the total cholesterol (*r*^2^ = 0.477, *p* = 0.001) while *Prevotellaceae_*NK3B31_group linearly predicted HbA1c levels (*r*^2^ = 0.463, *p* = 0.001; Figure 7 and Supplementary Table 4).

## Discussion

The prevalence of T2DM has been found to increase steadily at an earlier age among post-GDM women (Fugmann et al., 2015; Zhu and Zhang, 2016). Thus, it is crucial to elucidate a new potential predictor to discover the early onset of T2DM among post-GDM women. Recent studies have shown a positive interest in gut microbiota dysbiosis and GI in women with GDM (Kuang et al., 2017; Ferrocino et al., 2018; Ma et al., 2020). We used a novel approach to study the association between macronutrient intakes, metabolic phenotypes, and gut microbiota composition during the postpartum period in women with a previous GDM during pregnancy.

Our results indicated that Bacteroidetes was the predominant phyla in post-GDM women during postpartum, while the abundance of Firmicutes was lower in the GI group than in the NGT group. Fugmann et al. (2015) had explored the gut microbiota composition between 42 post-GDM women and 35 women without GDM at 3–16 months postpartum. Similarly, half of the post-GDM women have GI and the relative abundance of Firmicutes was lower in the post-GDM women (Fugmann et al., 2015). These findings resembling the gut microbiota profile of adults with T2DM, which suggests that gut microbiota dysbiosis may be a potential predictive biomarker of possible T2DM risks in post-GDM women (Larsen et al., 2010; Qin et al., 2012; Fugmann et al., 2015). However, Hasan et al. (2018) did not find significant differences in the gut microbiota composition among 60 post-GDM women and 68 women without a history of GDM after 5 years postpartum. By contrast, Zhang et al. (2013) found that the abundance of Firmicutes and *Clostridia* were more dominant in adults with T2DM than in the healthy controls (Zhang et al., 2013). Although we did not observe a significant difference in α and β diversity between the two groups, several gut microbial taxa exhibited a significant difference in abundance between GI and NGT groups. For instance, Proteobacteria and *Parasutterella* were primarily detected in the GI group. Proteobacteria is known to be a potential microbiota signature of dysbiosis and linked to obesity and T2DM (Shin et al., 2015; Nuli et al., 2019).

Interestingly, we have found nine moderate significant correlations between macronutrients, gut microbial taxa, and metabolic phenotypes using a Pearson’s correlation coefficient analysis. Besides, approximately 14 significant predictions of gut microbial taxa by macronutrients were identified using stepwise regression analysis. Results of the present study indicated that low protein, high cholesterol, and high monounsaturated fatty acids (MUFA) intakes moderately reduced the relative abundance of gut microbial taxa that belonged to Firmicutes in post-GDM women. Moreover, low fiber intakes and high total sugar significantly reduced the relative abundance of *Phascolarctobacterium*. *Phascolarctobacterium* is a member of Firmicutes and was reported to be correlated with cruciferous vegetable diet (Li et al., 2009). Wu et al. (2017) found *Phascolarctobacterium* had a high colonization rate and conferred benefits to host metabolism by producing short-chain fatty acids (SCFAs) (Zhang et al., 2015; Wu et al., 2017). Therefore, the imbalance in protein, cholesterol, MUFA, sugar, and fiber intakes may be responsible for the depletion of the relative abundance of Firmicutes, while also contributing to host dysregulation among post-GDM women with GI.

More importantly, approximately 13 predictions of metabolic phenotypes by gut microbial taxa were identified. The *Prevotellaceae_*NK3B31_group, which was significantly abundant in the GI group, was among the main gut microbiota linked to the elevation of FBG, HOMA-IR, and HbA1c levels. Egshatyan et al. (2016) noted that the relative abundance of *Prevotella* was positively correlated with carbohydrate intakes, and representation of *Prevotella* in the high-fat-protein cluster was found to be significantly associated with adults with insulin resistance (Egshatyan et al., 2016; Díaz-Rizzolo et al., 2020). In a study of gut microbiota composition of 41 pregnant women with GDM, Ferrocino et al. (2018) showed that *Prevotella* was linearly associated with HbA1c levels (Ferrocino et al., 2018). The presence or increased abundance of *Prevotellaceae*_NK3B31_group might have worsened GI in the GI group through their mucin-degrading properties, which may weaken the epithelial layer of the gut, promote metabolic endotoxemia, and trigger low-grade inflammation, leading to insulin resistance and hyperglycemia in women with GDM (Wright et al., 2000; Hasain et al., 2020).

The present study also found that lower MUFA and fiber intakes increased the relative abundance of *Lactobacillus*. Further analyses showed that the elevation of *Lactobacillus* and reduction of *Bifidobacterium* were associated with obesity. Consistent findings were observed in an earlier study that had involved 134 obese, 38 overweight, 76 lean, and 15 anorexic subjects (Million et al., 2013). Our study also indicated that elevation of *Lactobacillus* increased hs-CRP levels. Similarly, *Lactobacillus* species were predominant among 18 adults with T2DM and may be linked with low-grade inflammation (Larsen et al., 2010). Besides, *Lactobacillus* and *Ruminococcaceae*_UCG014 showed a linear relationship with postpartum 2HPP glucose levels. Similar associations were noted in studies involving 70 pregnant women who were obese and overweight (Gomez-Arango et al., 2016) and those in the first trimester who later developed GDM (Mokkala et al., 2017). We postulated that the elevation of these two bacteria might be related to a higher positive energy balance that leads to adiposity, which may trigger low-grade inflammation and attenuate insulin sensitivity causing GI in post-GDM women (Hasain et al., 2020).

Notable limitations associated with this study require further consideration. The main limitations are small sample size, high prevalence of GI, and lack of a healthy control group. The main objective of this study was to elucidate the relationship between diet, gut microbiota, and metabolic phenotypes in post-GDM women. *Post hoc* power analysis calculation using G^∗^Power analysis version 3.1.9.4 showed that the power analysis was sufficient (82.5%) to detect a significant correlation between diet and gut microbial taxa (Cohen’s effect size = 0.55) with a statistical significance of 0.05 and 24 samples. However, this number was small to compare the participant’s characteristics between GI and NGT groups. For example, the insufficient sample size might have caused a higher percentage of GI among post-GDM women at the early postpartum period. Besides, the majority of the recruited post-GDM women have pre-pregnancy obesity, required pharmacological intervention during pregnancy, and a lower percentage of exclusive breastfeeding was observed among post-GDM women with GI. These characteristics were the possible factors of persistent GI following a history of GDM (Benhalima et al., 2019). Moreover, assessment of both FBG and 2HPP blood glucose levels using a 2-h 75 g OGTT might have lead to a higher percentage of GI compared to assessment of FBG alone during the postpartum period (Lawrence et al., 2010). In addition, high early postpartum GI among post-GDM women may due to underlying undiagnosed diabetes before pregnancy (Mukerji et al., 2012). On the other hand, the α and β diversity of the gut microbiota between the two groups were not significant most probably because participants from both groups have recent history of GDM, small sample size, and no healthy women were included as the control group. Besides, it was not possible to demonstrate the gut microbiota shift from pregnancy to delivery as only single fecal samples were taken after delivery. Therefore, a larger sample size, inclusion of a healthy control group, and multiple fecal sampling at different pregnancy stages may provide a more significant comparison and would help to ascertain whether the findings could be adopted clinically. Moreover, some of the relationships between gut microbiota and host metabolism contradicted earlier studies, which was possibly due to microbiota being strain-specific. Consequently, the shotgun metagenomic approach is preferable because the 16S rRNA sequencing approach has limited robustness (Ranjan et al., 2016). Finally, although we observed a significant association between all parameters, we were unable to find a definitive causal relationship between the gut microbiota and GDM women because we employed a cross-sectional study design.

## Conclusion

Overall, macronutrient intakes were found to significantly preserved gut ecology and biochemical in post-GDM women. Macronutrient intakes may exert beneficial or detrimental effects on the gut microbial taxa and the host factors. We found that imbalance in the macronutrient intakes was associated with perturbation of pathogenic pathobionts, such as *Prevotellaceae*_NK3B31_group and *Lactobacillus* leading to metabolic dysregulation especially in the GI group. Therefore, dietary interventions or probiotics supplementation may be ideal for modulating gut microbiota composition and promoting good health outcomes among these women. However, the direction of crosstalk between diet and gut microbiota dysbiosis requires further validation and comparison. Thus, future research should include extensive longitudinal data and include multi-omics approaches, such as metagenomics, transcriptomics, proteomics, and metabolomics, which are warranted to determine diet-gut microbiota interactions as a potential preventive T2DM strategy in post-GDM women.

## Data Availability Statement

The datasets presented in this study can be found in online repositories: National Center for Biotechnology Information. The accession number for the raw sequence reads in the NCBI Sequence Read Archive (SRA) is SRP313726. The BioProject and BioSample accession numbers are PRJNA717644 and SAMN18499532–SAMN18499555, respectively.

## Ethics Statement

The studies involving human participants were reviewed and approved by the Universiti Kebangsaan’s Malaysia Human Research Ethics Committee (reference: UKM PPI/111/8/JEP-2018-022). The patients/participants provided their written informed consent to participate in this study.

## Author Contributions

NM and RR were the principal investigators and were responsible for the original ideas of the project. ZH conducted the experiments. ZH, SA, and KA analyzed the data and drafted the manuscript. ZH, KA, SA, NR, NM, RR, and EE-O revised and edited the manuscript. All authors have read and approved the final manuscript.

## Conflict of Interest

The authors declare that the research was conducted in the absence of any commercial or financial relationships that could be construed as a potential conflict of interest.

## Footnotes

1 https://en.novogene.com/
